# 2-[1-(1-Oxoindan-2-yl)eth­yl]indan-1-one

**DOI:** 10.1107/S1600536812029315

**Published:** 2012-07-04

**Authors:** Abdullah M. Asiri, Hassan M. Faidallah, Khalid A. Alamry, Seik Weng Ng, Edward R. T. Tiekink

**Affiliations:** a Center of Excellence for Advanced Materials Research (CEAMR), King Abdulaziz University, PO Box 80203, Jeddah 21589, Saudi Arabia; b Chemistry Department, Faculty of Science, King Abdulaziz University, PO Box 80203, Jeddah 21589, Saudi Arabia; c Department of Chemistry, University of Malaya, 50603 Kuala Lumpur, Malaysia

## Abstract

In the title compound, C_20_H_18_O_2_, the fused-ring systems are essentially planar (r.m.s. deviations of the nine fitted atoms = 0.009 and 0.027 Å) and exhibit an orthogonal relationship [dihedral angle = 79.83 (5)°]. To a first approximation, the ketone-O atoms are directed to opposite sides of the mol­ecule. A three-dimensional architecture arises in the crystal packing owing to C—H⋯O, C—H⋯π and π–π inter­actions [between centrosymmetrically related benzene rings with centroid–centroid distance = 3.7647 (10) Å].

## Related literature

For the biological activity of related indan-1-one derivatives, see: Vera-DiVaio *et al.* (2009 ▶). For a related structure see: Asiri *et al.* (2012 ▶).
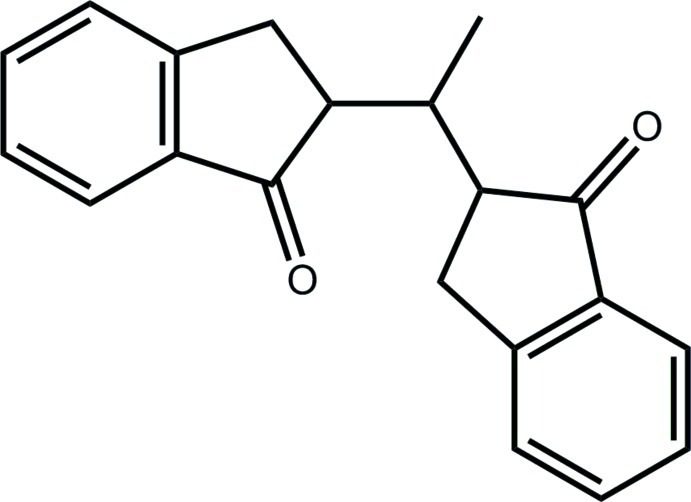


## Experimental

### 

#### Crystal data

C_20_H_18_O_2_
*M* *_r_* = 290.34
Triclinic, 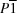

*a* = 7.9225 (6) Å
*b* = 10.1226 (8) Å
*c* = 10.3927 (7) Å
α = 103.200 (6)°
β = 103.304 (6)°
γ = 109.462 (7)°
*V* = 721.25 (9) Å^3^
*Z* = 2
Mo *K*α radiation
μ = 0.09 mm^−1^
*T* = 100 K
0.40 × 0.20 × 0.10 mm

#### Data collection

Agilent SuperNova Dual diffractometer with an Atlas detector
Absorption correction: multi-scan (*CrysAlis PRO*; Agilent, 2012 ▶) *T*_min_ = 0.748, *T*_max_ = 1.000
4961 measured reflections
3299 independent reflections
2663 reflections with *I* > 2σ(*I*)
*R*_int_ = 0.021

#### Refinement

*R*[*F* ^2^ > 2σ(*F* ^2^)] = 0.048
*wR*(*F* ^2^) = 0.130
*S* = 1.05
3299 reflections
199 parameters
H-atom parameters constrained
Δρ_max_ = 0.29 e Å^−3^
Δρ_min_ = −0.23 e Å^−3^

### 

Data collection: *CrysAlis PRO* (Agilent, 2012 ▶); cell refinement: *CrysAlis PRO*; data reduction: *CrysAlis PRO*; program(s) used to solve structure: *SHELXS97* (Sheldrick, 2008 ▶); program(s) used to refine structure: *SHELXL97* (Sheldrick, 2008 ▶); molecular graphics: *ORTEP-3 for Windows* (Farrugia, 1997 ▶) and *DIAMOND* (Brandenburg, 2006 ▶); software used to prepare material for publication: *publCIF* (Westrip, 2010 ▶).

## Supplementary Material

Crystal structure: contains datablock(s) global, I. DOI: 10.1107/S1600536812029315/mw2075sup1.cif

Structure factors: contains datablock(s) I. DOI: 10.1107/S1600536812029315/mw2075Isup2.hkl

Supplementary material file. DOI: 10.1107/S1600536812029315/mw2075Isup3.cml

Additional supplementary materials:  crystallographic information; 3D view; checkCIF report

## Figures and Tables

**Table 1:** Hydrogen-bond geometry (Å, °) *Cg*1 is the centroid of the C14–C19 benzene ring.

*D*—H⋯*A*	*D*—H	H⋯*A*	*D*⋯*A*	*D*—H⋯*A*
C12—H12⋯O1^i^	1.00	2.55	3.2317 (19)	125
C3—H3⋯*Cg*1^i^	0.95	2.62	3.5395 (17)	163
C11—H11*C*⋯*Cg*1^ii^	0.98	2.99	3.6481 (18)	126

Symmetry codes: (i) 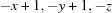; (ii) 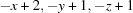.

**Fig. 1.**
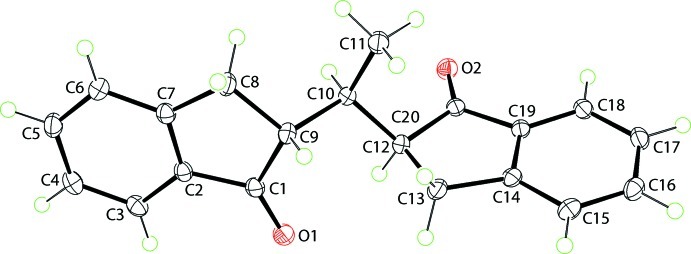
The molecular structure of (I) showing the atom-labelling scheme and displacement ellipsoids at the 50% probability level.

**Fig. 2.**
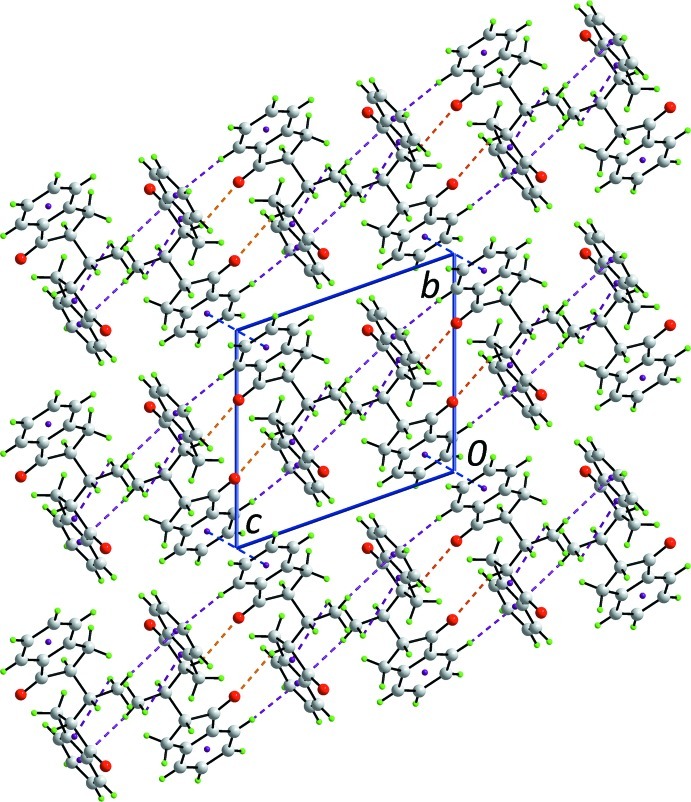
A view in projection down the a axis of the unit-cell contents of (I). The C—H···O, C—H···π and π—π interactions are shown as orange, purple and blue dashed lines, respectively.

## supplementary crystallographic information

### Comment

The motivation for the synthesis of the title compound, 2-[1-(1-oxo-2,3-dihydro-1*H*-inden-2-yl)ethyl]-2,3-dihydro-1*H*-inden-1-one (I), rests with its relationship to biologically active compounds (Vera-DiVaio *et al.*, 2009). Herein, the crystal and molecular structure of (I) is described in continuation of on-going structural studies of indan-1-one derivatives (Asiri *et al.*, 2012).

In (I), Fig. 1, each fused ring system is planar [C1-containing system: r.m.s. deviation of the nine fitted atoms = 0.009 Å with maximum deviation of 0.012 (2) Å for atom C7; C19-containing system: 0.027 Å and 0.044 (2) Å for atom C13]. The dihedral angle between the indan-1-one residues = 79.83 (5)°, indicating an almost orthogonal relationship. To a first approximation the ketone-O atoms are directed to opposite sides of the molecule.

In the crystal packing, molecules are arranged in a three-dimensional architecture by C—H···O and C—H···π interactions, Table 1, as well as π—π contacts between centrosymmetrically related C2–C6 benzene rings [inter-centroid distance = 3.7647 (10) Å for symmetry operation -*x*, -*y*, -*z*], Fig. 2.

### Experimental

A solution of acetaldehyde (0.44 g, 0.01 *M*) in ethanol (20 mL) was added to a stirred solution of 1-indanone (1.3 g,0.01 *M*) in (20%) ethanolic KOH (20 mL) and stirring was maintained at room temperature for 6 h. The reaction mixture was then poured onto water (200 mL) and set aside overnight. The precipitated solid product was collected by filtration, washed with water, dried and recrystallized from ethanol. *M.P.*: 413–414 K. Yield: 86%.

### Refinement

Carbon-bound H-atoms were placed in calculated positions [C—H = 0.95–0.99 Å, *U*_iso_(H) = 1.2–1.5*U*_eq_(C)] and were included in the refinement in the riding model approximation. Owing to poor agreement, the (-8 1 5) reflection was omitted from the final cycles of refinement.

### Crystal data

**Table:**

C_20_H_18_O_2_	*Z* = 2
*M**_r_* = 290.34	*F*(000) = 308
Triclinic, *P*1	*D*_x_ = 1.337 Mg m^−^^3^
Hall symbol: -P 1	Mo *K*α radiation, λ = 0.71073 Å
*a* = 7.9225 (6) Å	Cell parameters from 2265 reflections
*b* = 10.1226 (8) Å	θ = 2.3–27.5°
*c* = 10.3927 (7) Å	µ = 0.09 mm^−^^1^
α = 103.200 (6)°	*T* = 100 K
β = 103.304 (6)°	Prism, colourless
γ = 109.462 (7)°	0.40 × 0.20 × 0.10 mm
*V* = 721.25 (9) Å^3^	

### Data collection

**Table:**

Agilent SuperNova Dual diffractometer with an Atlas detector	3299 independent reflections
Radiation source: SuperNova (Mo) X-ray Source	2663 reflections with *I* > 2σ(*I*)
Mirror monochromator	*R*_int_ = 0.021
Detector resolution: 10.4041 pixels mm^-1^	θ_max_ = 27.6°, θ_min_ = 2.3°
ω scan	*h* = −10→8
Absorption correction: multi-scan (*CrysAlis PRO*; Agilent, 2012)	*k* = −13→13
*T*_min_ = 0.748, *T*_max_ = 1.000	*l* = −13→13
4961 measured reflections	

### Refinement

**Table:**

Refinement on *F*^2^	Primary atom site location: structure-invariant direct methods
Least-squares matrix: full	Secondary atom site location: difference Fourier map
*R*[*F*^2^ > 2σ(*F*^2^)] = 0.048	Hydrogen site location: inferred from neighbouring sites
*wR*(*F*^2^) = 0.130	H-atom parameters constrained
*S* = 1.05	*w* = 1/[σ^2^(*F*_o_^2^) + (0.0616*P*)^2^ + 0.1751*P*] where *P* = (*F*_o_^2^ + 2*F*_c_^2^)/3
3299 reflections	(Δ/σ)_max_ < 0.001
199 parameters	Δρ_max_ = 0.29 e Å^−^^3^
0 restraints	Δρ_min_ = −0.23 e Å^−^^3^

### Special details

**Table:**

Geometry. All e.s.d.'s (except the e.s.d. in the dihedral angle between two l.s. planes) are estimated using the full covariance matrix. The cell e.s.d.'s are taken into account individually in the estimation of e.s.d.'s in distances, angles and torsion angles; correlations between e.s.d.'s in cell parameters are only used when they are defined by crystal symmetry. An approximate (isotropic) treatment of cell e.s.d.'s is used for estimating e.s.d.'s involving l.s. planes.
Refinement. Refinement of *F*^2^ against ALL reflections. The weighted *R*-factor *wR* and goodness of fit *S* are based on *F*^2^, conventional *R*-factors *R* are based on *F*, with *F* set to zero for negative *F*^2^. The threshold expression of *F*^2^ > σ(*F*^2^) is used only for calculating *R*-factors(gt) *etc*. and is not relevant to the choice of reflections for refinement. *R*-factors based on *F*^2^ are statistically about twice as large as those based on *F*, and *R*- factors based on ALL data will be even larger.

### Fractional atomic coordinates and isotropic or equivalent isotropic displacement parameters (Å^2^)

**Table:**

	*x*	*y*	*z*	*U*_iso_*/*U*_eq_	
O1	0.33484 (17)	0.32248 (12)	0.00936 (11)	0.0238 (3)	
O2	0.79274 (16)	0.78862 (12)	0.39844 (11)	0.0198 (3)	
C1	0.3010 (2)	0.27906 (16)	0.10437 (15)	0.0163 (3)	
C2	0.1106 (2)	0.20108 (16)	0.11050 (15)	0.0155 (3)	
C3	−0.0656 (2)	0.16295 (17)	0.01190 (16)	0.0187 (3)	
H3	−0.0744	0.1886	−0.0711	0.022*	
C4	−0.2270 (2)	0.08665 (17)	0.03875 (17)	0.0210 (4)	
H4	−0.3488	0.0593	−0.0265	0.025*	
C5	−0.2120 (2)	0.04949 (18)	0.16127 (17)	0.0214 (4)	
H5	−0.3244	−0.0030	0.1779	0.026*	
C6	−0.0367 (2)	0.08758 (17)	0.25916 (17)	0.0195 (3)	
H6	−0.0280	0.0619	0.3421	0.023*	
C7	0.1266 (2)	0.16466 (16)	0.23241 (16)	0.0160 (3)	
C8	0.3313 (2)	0.21549 (18)	0.31939 (16)	0.0187 (3)	
H8A	0.3590	0.1297	0.3313	0.022*	
H8B	0.3606	0.2859	0.4132	0.022*	
C9	0.4496 (2)	0.29281 (16)	0.23705 (15)	0.0159 (3)	
H9	0.5224	0.2343	0.2081	0.019*	
C10	0.5928 (2)	0.45371 (16)	0.32567 (15)	0.0144 (3)	
H10	0.5205	0.5090	0.3618	0.017*	
C11	0.7368 (2)	0.45252 (18)	0.45280 (16)	0.0200 (3)	
H11A	0.8267	0.5547	0.5091	0.030*	
H11B	0.8060	0.3952	0.4201	0.030*	
H11C	0.6694	0.4070	0.5101	0.030*	
C12	0.6953 (2)	0.53610 (16)	0.23973 (15)	0.0161 (3)	
H12	0.5969	0.5394	0.1621	0.019*	
C13	0.8140 (2)	0.46661 (17)	0.17407 (16)	0.0190 (3)	
H13A	0.8209	0.3838	0.2074	0.023*	
H13B	0.7582	0.4284	0.0705	0.023*	
C14	1.0084 (2)	0.59087 (16)	0.22124 (15)	0.0155 (3)	
C15	1.1668 (2)	0.58854 (17)	0.18552 (16)	0.0183 (3)	
H15	1.1600	0.5013	0.1221	0.022*	
C16	1.3348 (2)	0.71586 (18)	0.24424 (16)	0.0197 (3)	
H16	1.4434	0.7157	0.2201	0.024*	
C17	1.3462 (2)	0.84420 (18)	0.33833 (16)	0.0191 (3)	
H17	1.4630	0.9296	0.3789	0.023*	
C18	1.1889 (2)	0.84811 (17)	0.37301 (16)	0.0169 (3)	
H18	1.1957	0.9353	0.4363	0.020*	
C19	1.0201 (2)	0.72010 (16)	0.31216 (15)	0.0149 (3)	
C20	0.8326 (2)	0.69643 (16)	0.32874 (15)	0.0153 (3)	

### Atomic displacement parameters (Å^2^)

**Table:**

	*U*^11^	*U*^22^	*U*^33^	*U*^12^	*U*^13^	*U*^23^
O1	0.0241 (6)	0.0235 (6)	0.0171 (5)	0.0014 (5)	0.0050 (5)	0.0092 (5)
O2	0.0198 (6)	0.0187 (6)	0.0208 (6)	0.0077 (5)	0.0085 (5)	0.0046 (5)
C1	0.0184 (8)	0.0119 (7)	0.0156 (7)	0.0037 (6)	0.0052 (6)	0.0038 (6)
C2	0.0155 (8)	0.0116 (7)	0.0170 (7)	0.0044 (6)	0.0046 (6)	0.0031 (6)
C3	0.0201 (8)	0.0154 (7)	0.0175 (7)	0.0062 (6)	0.0028 (6)	0.0047 (6)
C4	0.0155 (8)	0.0192 (8)	0.0226 (8)	0.0054 (7)	0.0016 (6)	0.0040 (7)
C5	0.0154 (8)	0.0204 (8)	0.0257 (8)	0.0041 (6)	0.0086 (7)	0.0059 (7)
C6	0.0188 (8)	0.0198 (8)	0.0203 (7)	0.0062 (7)	0.0083 (6)	0.0081 (7)
C7	0.0160 (8)	0.0133 (7)	0.0175 (7)	0.0055 (6)	0.0053 (6)	0.0038 (6)
C8	0.0152 (8)	0.0199 (8)	0.0206 (7)	0.0043 (6)	0.0056 (6)	0.0103 (7)
C9	0.0141 (7)	0.0155 (7)	0.0183 (7)	0.0047 (6)	0.0064 (6)	0.0070 (6)
C10	0.0136 (7)	0.0151 (7)	0.0139 (7)	0.0045 (6)	0.0050 (6)	0.0052 (6)
C11	0.0167 (8)	0.0238 (8)	0.0193 (7)	0.0064 (7)	0.0050 (6)	0.0104 (7)
C12	0.0145 (8)	0.0159 (7)	0.0151 (7)	0.0029 (6)	0.0051 (6)	0.0050 (6)
C13	0.0200 (8)	0.0156 (7)	0.0198 (7)	0.0038 (6)	0.0107 (6)	0.0038 (6)
C14	0.0175 (8)	0.0152 (7)	0.0147 (7)	0.0054 (6)	0.0063 (6)	0.0073 (6)
C15	0.0224 (8)	0.0186 (8)	0.0201 (7)	0.0108 (7)	0.0106 (7)	0.0102 (7)
C16	0.0165 (8)	0.0267 (8)	0.0213 (7)	0.0116 (7)	0.0080 (6)	0.0120 (7)
C17	0.0134 (8)	0.0205 (8)	0.0191 (7)	0.0040 (6)	0.0020 (6)	0.0069 (7)
C18	0.0161 (8)	0.0167 (7)	0.0160 (7)	0.0054 (6)	0.0039 (6)	0.0054 (6)
C19	0.0143 (8)	0.0164 (7)	0.0144 (7)	0.0053 (6)	0.0042 (6)	0.0076 (6)
C20	0.0156 (8)	0.0164 (7)	0.0130 (7)	0.0046 (6)	0.0040 (6)	0.0070 (6)

### Geometric parameters (Å, º)

**Table:**

O1—C1	1.2172 (18)	C10—H10	1.0000
O2—C20	1.2200 (18)	C11—H11A	0.9800
C1—C2	1.475 (2)	C11—H11B	0.9800
C1—C9	1.537 (2)	C11—H11C	0.9800
C2—C7	1.388 (2)	C12—C20	1.532 (2)
C2—C3	1.397 (2)	C12—C13	1.537 (2)
C3—C4	1.383 (2)	C12—H12	1.0000
C3—H3	0.9500	C13—C14	1.508 (2)
C4—C5	1.399 (2)	C13—H13A	0.9900
C4—H4	0.9500	C13—H13B	0.9900
C5—C6	1.388 (2)	C14—C19	1.388 (2)
C5—H5	0.9500	C14—C15	1.394 (2)
C6—C7	1.397 (2)	C15—C16	1.389 (2)
C6—H6	0.9500	C15—H15	0.9500
C7—C8	1.506 (2)	C16—C17	1.398 (2)
C8—C9	1.552 (2)	C16—H16	0.9500
C8—H8A	0.9900	C17—C18	1.384 (2)
C8—H8B	0.9900	C17—H17	0.9500
C9—C10	1.546 (2)	C18—C19	1.396 (2)
C9—H9	1.0000	C18—H18	0.9500
C10—C11	1.539 (2)	C19—C20	1.481 (2)
C10—C12	1.541 (2)		
			
O1—C1—C2	125.93 (14)	C10—C11—H11A	109.5
O1—C1—C9	125.80 (14)	C10—C11—H11B	109.5
C2—C1—C9	108.26 (12)	H11A—C11—H11B	109.5
C7—C2—C3	121.93 (15)	C10—C11—H11C	109.5
C7—C2—C1	109.99 (13)	H11A—C11—H11C	109.5
C3—C2—C1	128.07 (14)	H11B—C11—H11C	109.5
C4—C3—C2	117.95 (14)	C20—C12—C13	105.56 (12)
C4—C3—H3	121.0	C20—C12—C10	111.74 (12)
C2—C3—H3	121.0	C13—C12—C10	115.59 (12)
C3—C4—C5	120.44 (15)	C20—C12—H12	107.9
C3—C4—H4	119.8	C13—C12—H12	107.9
C5—C4—H4	119.8	C10—C12—H12	107.9
C6—C5—C4	121.51 (15)	C14—C13—C12	105.17 (12)
C6—C5—H5	119.2	C14—C13—H13A	110.7
C4—C5—H5	119.2	C12—C13—H13A	110.7
C5—C6—C7	118.21 (14)	C14—C13—H13B	110.7
C5—C6—H6	120.9	C12—C13—H13B	110.7
C7—C6—H6	120.9	H13A—C13—H13B	108.8
C2—C7—C6	119.95 (14)	C19—C14—C15	119.78 (14)
C2—C7—C8	111.50 (14)	C19—C14—C13	111.79 (14)
C6—C7—C8	128.54 (14)	C15—C14—C13	128.43 (14)
C7—C8—C9	105.61 (12)	C16—C15—C14	118.87 (15)
C7—C8—H8A	110.6	C16—C15—H15	120.6
C9—C8—H8A	110.6	C14—C15—H15	120.6
C7—C8—H8B	110.6	C15—C16—C17	120.85 (15)
C9—C8—H8B	110.6	C15—C16—H16	119.6
H8A—C8—H8B	108.8	C17—C16—H16	119.6
C1—C9—C10	114.59 (12)	C18—C17—C16	120.66 (15)
C1—C9—C8	104.64 (12)	C18—C17—H17	119.7
C10—C9—C8	112.64 (13)	C16—C17—H17	119.7
C1—C9—H9	108.2	C17—C18—C19	118.06 (14)
C10—C9—H9	108.2	C17—C18—H18	121.0
C8—C9—H9	108.2	C19—C18—H18	121.0
C11—C10—C12	110.83 (12)	C14—C19—C18	121.75 (15)
C11—C10—C9	109.67 (12)	C14—C19—C20	109.52 (13)
C12—C10—C9	112.68 (12)	C18—C19—C20	128.74 (14)
C11—C10—H10	107.8	O2—C20—C19	126.32 (14)
C12—C10—H10	107.8	O2—C20—C12	125.82 (14)
C9—C10—H10	107.8	C19—C20—C12	107.85 (12)

### Hydrogen-bond geometry (Å, º)

Cg1 is the centroid of the C14–C19 benzene ring.

**Table:**

*D*—H···*A*	*D*—H	H···*A*	*D*···*A*	*D*—H···*A*
C12—H12···O1^i^	1.00	2.55	3.2317 (19)	125
C3—H3···*Cg*1^i^	0.95	2.62	3.5395 (17)	163
C11—H11*C*···*Cg*1^ii^	0.98	2.99	3.6481 (18)	126

Symmetry codes: (i) −*x*+1, −*y*+1, −*z*; (ii) −*x*+2, −*y*+1, −*z*+1.
